# *Vibrio cholerae vexH* Encodes a Multiple Drug Efflux Pump That Contributes to the Production of Cholera Toxin and the Toxin Co-Regulated Pilus

**DOI:** 10.1371/journal.pone.0038208

**Published:** 2012-05-30

**Authors:** Dawn L. Taylor, Xiaowen R. Bina, James E. Bina

**Affiliations:** University of Pittsburgh School of Medicine, Department of Microbiology and Molecular Genetics, Pittsburgh, Pennsylvania, United States of America; University of Malaya, Malaysia

## Abstract

The resistance-nodulation-division (RND) efflux systems are ubiquitous transporters that function in antimicrobial resistance. Recent studies showed that RND systems were required for virulence factor production in *Vibrio cholerae*. The *V. cholerae* genome encodes six RND efflux systems. Three of the RND systems (VexB, VexD, and VexK) were previously shown to be redundant for in *vitro* resistance to bile acids and detergents. A mutant lacking the VexB, VexD, and VexK RND pumps produced wild-type levels of cholera toxin (CT) and the toxin co-regulated pilus (TCP) and was moderately attenuated for intestinal colonization. In contrast, a RND negative mutant produced significantly reduced amounts of CT and TCP and displayed a severe colonization defect. This suggested that one or more of the three uncharacterized RND efflux systems (i.e. VexF, VexH, and VexM) were required for pathogenesis. In this study, a genetic approach was used to generate a panel of *V. cholerae* RND efflux pump mutants in order to determine the function of VexH in antimicrobial resistance, virulence factor production, and intestinal colonization. VexH contributed to *in vitro* antimicrobial resistance and exhibited a broad substrate specificity that was redundant with the VexB, VexD, and VexK RND efflux pumps. These four efflux pumps were responsible for *in vitro* antimicrobial resistance and were required for virulence factor production and intestinal colonization. Mutation of the VexF and/or VexM efflux pumps did not affect *in vitro* antimicrobial resistance, but did negatively affect CT and TCP production. Collectively, our results demonstrate that the *V. cholerae* RND efflux pumps have redundant functions in antimicrobial resistance and virulence factor production. This suggests that the RND efflux systems contribute to *V. cholerae* pathogenesis by providing the bacterium with protection against antimicrobial compounds that are present in the host and by contributing to the regulated expression of virulence factors.

## Introduction

*Vibrio cholerae* is a Gram negative, motile, facultative anaerobic bacterium, and the causative agent of cholera, a severe diarrhoeal disease, which untreated can rapidly lead to dehydration, hypotensive shock, and death. *V. cholerae* is a common inhabitant of aquatic environments where it can survive and persist in association with aquatic plants and animals. Humans acquire cholera by ingesting *V. cholerae* contaminated food or water [1]. Upon ingestion, *V. cholerae* colonizes the small intestine where a complex regulatory cascade is induced, resulting in the production of several important virulence factors including cholera toxin (CT) and the toxin co-regulated pilus (TCP) [2], [3]. CT is an AB-type enterotoxin that is responsible for the secretory diarrhoea that is characteristic of cholera [4]. The TCP is a type IV bundle forming pilus that is essential for intestinal colonization of both humans and laboratory animals [2], [5]–[7]. CT and TCP production are tightly controlled by a hierarchical regulatory system called the ToxR regulon [8], [9]. In response to unknown stimuli, ToxR and TcpP, two membrane associated transcriptional regulators, activate transcription of *toxT* [3], [10]–[12]. ToxT, an AraC-family transcriptional regulator, directly activates the expression of the *ctxAB* and the *tcpA-F* operons which encode for the production of CT and the TCP, plus a number of accessory virulence genes [13]–[15].

In order to colonize and survive in the host, *V. cholerae* must protect itself from the toxic effects of antimicrobial compounds that are present in the gastrointestinal tract (GI). *V. cholerae* does this by limiting the uptake and intracellular accumulation of toxic antimicrobial molecules that are present in the GI tract. This is accomplished by modulating the outer membrane permeability (e.g. through the production of porin proteins and cell envelope modifications) in conjunction with efflux of the antimicrobial molecules via active efflux transporters [16]–[20]. There are five different active efflux systems described in bacteria: the ATP-binding cassette superfamily (ABC), the small multidrug resistance family (SMR), the multi antimicrobial extrusion protein family (MATE), the major facilitator superfamily (MFS), and the resistance-nodulation-cell division superfamily (RND) [21]. The RND family is particularly interesting because of its broad substrate specificity and its association with multidrug resistance in many Gram negative pathogens. Individual RND efflux systems, including the *V. cholerae* VexAB-TolC [22], *Escherichia coli* AcrAB-TolC [23], and *Pseudomonas aeruginosa* MexAB-OprM systems [24], have been shown to efflux chemically diverse antimicrobial compounds including: dyes, detergents, antibiotics, and antimicrobial peptides [25].

RND efflux systems are tripartite transporters that function as proton-substrate antiporters [26], [27]. RND efflux systems are composed of an outer membrane pore protein (OMP) that is homologous to *E. coli tolC,* a periplasmic membrane fusion protein (MFP), and an integral cytoplasmic membrane pump protein belonging to the RND superfamily of transporters [27]–[31]. These three components function to form a channel for the extrusion of substrates from within the cell envelope to the external environment. Most Gram negative pathogens encode multiple RND efflux systems; *V. cholerae* encodes six. In *V. cholerae*, each RND system is separately encoded in an operon structure wherein the RND efflux pump protein has at least one associated MFP whose gene is located upstream of the pump gene. It appears that all six RND efflux systems share the same TolC OMP which is encoded separately on the chromosome [32]. Previous work in our laboratory showed that three of the RND efflux pumps (VexB, VexD, and VexK) were required for antimicrobial resistance *in vitro*. The VexB RND efflux pump exhibited a very broad substrate specificity and contributed resistance to bile acids, detergents, and several antibiotics. In contrast, the VexD and VexK RND pumps appeared to only efflux bile acids and detergents, respectively [22], [32].

Recently, our laboratory reported that the *V. cholerae* RND efflux systems were not only important for antimicrobial resistance and intestinal colonization, but were also important for CT and TCP production [32]. A mutant that lacked all six RND efflux pumps (i.e. ΔRND) was attenuated for CT and TCP production and was hypersensitive to antibiotics. Although the VexB, VexD, and VexK efflux pumps contributed to *in vitro* antimicrobial resistance, a mutant lacking the *vexBDK* genes produced WT levels of CT and TcpA. This finding suggested that one or more of the three remaining RND pumps (VexF, VexH, and VexM) must function in virulence factor production. In this study we have further characterized these three RND efflux pumps. Using a genetic approach to generate mutant strains with the RND efflux pumps deleted in various permutations, we found that the VexH RND efflux pump contributed to antimicrobial resistance, CT and TCP production, and successful colonization of the infant mouse small intestine. VexF and VexM did not appear to function in antimicrobial resistance *in vitro*, but were required for high level production of CT and TCP.

## Materials and Methods

### Ethics Statement

This study was performed in strict accordance with the recommendations in the Guide for the Care and Use of Laboratory Animals of the National Institutes of Health. The animal protocol (#1505R2) was approved by the Institutional Animal Care and Use Committee of the University of Tennessee Health Science Center.

### Strains and Growth Conditions

Bacterial strains and plasmids used in this study are listed in Table 1. *E. coli* strain EC100D*pir*+ was used for all cloning experiments. *E. coli* strain SM10λpir [33] was used for conjugation of plasmids into *V. cholerae*. All *V. cholerae* strains used in this study were derivatives of O1 El Tor strain N16961 [34]. *V. cholerae* strains N16961 and N16961 Δ*lacZ* were used as the wild-type (WT) control strains in all experiments. All bacterial strains were grown in Luria-Bertani (LB) broth or on LB agar at 37°C. *V. cholerae* was grown in AKI broth under AKI growth conditions for the CT and TCP bioassays [35]. AKI growth conditions were as follows: a fresh saturated overnight LB broth culture of the indicated strain was inoculated 1∶10,000 into 10 mL of AKI broth in a 18×175 mm test tube. The test tube was then incubated statically at 37°C for four hours when the broth culture was transferred into a sterile 125 mL Erlenmeyer flask. The Erlenmeyer flask was incubated with shaking overnight before CT and TCP production was assessed. Bacterial stocks were maintained at −80°C in LB broth containing 25% glycerol. Growth media was supplemented with carbenicillin (Cb) and streptomycin (Sm) at 100 µg/mL when required.

**Table 1:** Bacterial strains, Plasmids, and Oligonucleotides.

Strain	Genotype	Strain #	Source
*Vibrio cholerae*			
N16961-Sm	Spontaneous Streptomycin-resistant 01 El Tor strain N16961 Δ*lacZ*	JB58	[22]
Δ*vexH*	N16961-Sm Δ*vexH*	JB116	[32]
Δ*vexDH*	N16961-Sm Δ*vexD* Δ*vexH*	JB186	[32]
Δ*vexDHM*	N16961-Sm Δ*vexD* Δ*vexH* Δ*vexM*	JB386	[32]
Δ*vexDF*	N16961-Sm Δ*vexD* Δ*vexF*	JB435	[32]
Δ*vexDFHM*	N16961-Sm Δ*vexD* Δ*vexF* Δ*vexH* Δ*vexM*	JB459	[32]
Δ*vexDFHKM*	N16961-Sm Δ*vexD* Δ*vexF* Δ*vexH* Δ*vexK* Δ*vexM*	JB464	[32]
Δ*RND*	N16961-Sm Δ*vexB* Δ*vexD* Δ*vexF* Δ*vexH* Δ*vexK* Δ*vexM*	JB485	[32]
Δ*vexB*	N16961-Sm Δ*vexB*	JB495	[22]
Δ*vexK*	N16961-Sm Δ*vexK*	JB528	[32]
Δ*vexBK*	N16961-Sm Δ*vexB* Δ*vexK*	JB531	[32]
Δ*vexD*	N16961-Sm Δ*vexD*	JB692	[22]
Δ*vexBD*	N16961-Sm Δ*vexB* Δ*vexD*	JB694	[22]
Δ*vexBDHK*	N16961-Sm Δ*vexB* Δ*vexD* Δ*vexH* Δ*vexK*	DT12	This study
Δ*vexBHK*	N16961-Sm Δ*vexB* Δ*vexH* Δ*vexK*	DT23	This study
Δ*vexBDH*	N16961-Sm Δ*vexB* Δ*vexD* Δ*vexH*	DT30	This study
Δ*vexBH*	N16961-Sm Δ*vexB* Δ*vexH*	DT60	This study
Δ*vexHK*	N16961-Sm Δ*vexH* Δ*vexK*	DT64	This study
Δ*vexDK*	N16961-Sm Δ*vexD* Δ*vexK*	DT70	This study
Δ*vexDHK*	N16961-Sm Δ*vexD* Δ*vexH* Δ*vexK*	DT76	This study
*Escherichia coli*			
EC100D*pir+*	*F^-^ mcrA* Δ *(mrr-hsdRMS-mcrBC) Φ80dlacZ*Δ*M15* Δ*lacX74 recA1 endA1 araD139* Δ *(ara, leu)7697 galU galK λ^-^ rpsL (Str^R^) nupG pir+*		Epicentre
SM10λ*pir*	*thi-1 thr leu tonA lacY supE recA::*RP4-2-4-Tc::Mu Km^r^ (λ *pir*R6K)		[64]
Plasmids			
pWM91	Suicide plasmid vector used for allelic exchange		[65]
pM132	pWM91:: ΔVC0914		[32]
pM133	pWM91:: ΔVC1673		[32]

### Chemicals and Enzymes

Enzymes for cloning experiments were purchased from New England Biolabs (Beverly, MA). Bacterial growth media was purchased from Difco (Lawrence, KS) and chemicals were purchased from Sigma-Aldrich (St Louis, MO).

### Mutant Construction

Unmarked in-frame deletions of the RND efflux pump protein gene in each respective strain was constructed by allelic exchange using genetic constructs and methods that have been previously described [22], [32]. The Δ*vexBDHK*, Δ*vexBHK*, Δ*vexBDH*, and Δ*vexBH* mutants were derived by deletion of *vexH* in strains JB740, JB531, JB694, and JB495, respectively. The Δ*vexHK*, Δ*vexDK*, and Δ*vexDHK* mutants were derived by deletion of *vexK* deletion in strains JB116, JB692, and JB186, respectively.

### Antimicrobial Susceptibility Assays

Antimicrobial susceptibility tests were performed using antibiotic and detergent gradient agar plates as previously described [32], [36]. Each 9×9 cm gradient plate was inoculated with six strains, including N16961 and ΔRND which served as internal controls, before being incubated at 37°C. The following day the length of bacterial growth along the antimicrobial gradient was recorded for each strain. Reported values represent the average from a minimum of three independent experiments.

### CT and TCP Quantification

CT and TCP production were assayed as previously described [32] from cultures grown under AKI growth conditions. CT was quantified using a GM_1_ ganglioside ELISA. TCP production was quantified by Western immunoblotting using a polyclonal antibody that was directed towards TcpA, the pilin subunit of the TCP [32]. The polyclonal antisera against CT and TcpA were kindly provided by John Mekalanos (Harvard Medical School, Boston, MA).

### Growth Analysis in the Infant Mouse Model

The colonization phenotype of the RND efflux mutants were assessed using the infant mouse competition assay as previously described [32], [37]. Briefly, 5–7 day old mice were separated from their mothers 2 h prior to inoculation. The infant mice were then anaesthetized with isoflurane (Aceto Pharm, NY) and inoculated by gavage using a 30 cm length of 0.011″ ×0.024″ polyethylene tubing that was attached via a 30.5 GA needle to a 1 cc syringe containing the inoculum. The inoculum consisted of a mixture of the wild-type strain (*lacZ*+) and the RND mutant strain (*lacZ*-) at a 1∶1 or 1∶100 ratio (WT:mutant) and administered in a 50 µL volume that contained ∼2.5×10^4^ cfu of each strain; for the 1∶100 inoculum the mutant titer was increased to ∼2.5×10^6^ cfu. An aliquot of the inoculum was also serially diluted and spread onto LB plates containing Sm and X-gal to verify the input ratio. Following inoculation the mice were kept in a humidified incubator at 30°C. The following day, the infected mice were sacrificed and the small intestine was removed from above the cecum and homogenized in 5 mL of sterile phosphate buffered saline. Serial dilutions of the homogenates were then spread onto LB agar plates containing Sm and X-gal. The agar plates were then incubated at 37°C overnight when the resulting bacterial colonies were quantified as WT (*lacZ*+) or mutant (*lacZ*-) based on colony color. A competitive index (CI) was calculated for each mutant strain as the ratio of the WT to the mutant in the input inoculum divided by the ratio of the WT to mutant in the output from the mouse intestinal homogenates. To determine the in vitro competitive index, an inoculum consisting of a 1∶1 ratio of the test and control strain was inoculated into fresh LB or media and cultured with shaking overnight at 37°C before being serially diluted and spread onto LB plates containing Sm and X-gal to determine the output ratio. Standard bacteria growth assays in M9-glycerol minimal media were also performed to control for potential unknown metabolic differences that could affect growth of the mutants in vivo. A theoretical CI was calculated for mutant strains that could not be recovered from the mouse challenge experiments by using an artificial value of 1 recovered cfu for each strain.

The infant mouse colonization assay was performed identical to the competition assay with the exception that the inoculum consisted of a single strain of *V. cholerae* with the mice receiving either 2×10^6^ or 2×10^8^ cfu. Following overnight incubation, the intestinal homogenates were serially diluted before being spread onto LB-Sm agar plates to enumerate the bacterial loads in the small intestine of each mouse.

## Results

### Function of VexH in Antimicrobial Resistance

Deletion of *vexH* alone did not affect *V. cholerae* susceptibility to any of the tested antimicrobial compounds (Table 2). This is consistent with the reported functional redundancy among the *V. cholerae* RND efflux pumps [32]. Deletion of *vexH* in a Δ*vexB* background resulted in increased sensitivity to Triton X-100, ampicillin and novobiocin suggesting that this detergent and these antibiotics were substrates for the VexH RND efflux pump. This finding was corroborated by the corresponding increase in susceptibility to ampicillin and novobiocin in the Δ*vexBDKH* strain relative to the parental Δ*vexBDK* strain (Table 2). Novobiocin was also found to be a substrate for the VexB and VexK RND efflux systems as evidenced by the increased susceptibility observed for these mutants.

**Table 2:** Antimicrobial susceptibility of *V. cholerae* RND mutants.

	Mean length of mutant growth relative to WT (s.d.)^1^							
	Cholate		Deoxycholate		Triton X-100	Novobiocin	Ampicillin	
Strain	5%	0.05%	3%	0.01%	0.01%	0.6 µg/mL	10 µg/mL	2 µg/mL
N16961-Sm	100(0)	100(0)	100(0)	100(0)	100(0)	100(0)	100(0)	100(0)
Δ*vexB*	100(0)	100(0)	100(0)	100(0)	34.4(±3.1)†	66.7(±25.5)†	22.9(±7.8)†	100(0)
Δ*vexH*	100(0)	100(0)	100(0)	100(0)	100(0)	75.7(±29.4)	100(0)	100(0)
Δ*vexBD*	NG	58.9(±14.1)†	NG	73.9(±5.5)†	35.6(±1.6)†	58.9(±13.5)†	40.0(±14.6)†	100(0)
Δ*vexBH*	100(0)	100(0)	100(0)	100(0)	24.0(±4.3)*	19.3(±2.7)*	NG	20.6(±3.9)*
Δ*vexBK*	100(0)	100(0)	100(0)	100(0)	23.9(±4.8)*	23.2(±10.5)*	22.0(±10.2)†	100(0)
Δ*vexBDH*	NG	31.1(±1.6) 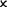 ¡	NG	47.2(±0.1) 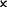 ¡	26.4(±8.2)	19.3(±2.6)	NG	20.7(±1.7)*
Δ*vexBDK*	NG	38.9(±1.6) 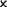 ¡	NG	47.2(±0.1) 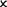 ¡	23.1(±4.8)	24.6(±9.5)	23.8(±10.4)†	100(0)
Δ*vexBHK*	100(0)	100(0)	100(0)	100(0)	24.3(±4.6)	8.2(±8.9)	NG	22.0(±1.1)*
Δ*vexDHK*	34.4(±1.6)†	100(0)	100(0)	100(0)	100(0)	62.2(±31.8)	100(0)	100(0)
Δ*vexBDHK*	NG	25.9(±3.32)	NG	33.3(±8.9)	23.6(±3.3)	12.8(±5.4)	NG	19.2(±2.3)*
Δ*RND*	NG	25.9(±3.32)	NG	34.8(±6.8)	23.8(±5.9)	10.2(±8.8)	NG	23(±2.9)*

Antimicrobial susceptibility was determined using antibiotic and detergent gradient agar plates.

1 The length of the mutant bacterial growth across the 90×90 mm gradient plate normalized to 100 mm. Data represents the average of three or more experiments with the standard deviation in parenthesis. Unpaired t-test was used to determine significance.

† p<0.001 compared to N16961-Sm;

*p<0.05 compared to Δ*vexB*;

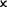
p<0.05 compared to Δ*vexBD*;

¡p<0.05 compared to Δ*vexBDHK*. NG: no bacterial growth.

VexB and VexD were previously shown to efflux bile salts. Therefore *vexH* was deleted in the Δ*vexBD* background in order to test if *vexH* contributed to bile salt resistance. The resulting Δ*vexBDH* mutant exhibited an increase in susceptibility to cholate and deoxycholate (Table 2). A similar increase in bile salt susceptibility was observed following the introduction of the *vexH* deletion into a Δ*vexBDK* background. These results support the conclusion that bile salts were substrates for the VexH RND efflux pump. The observation that the cholate and deoxycholate susceptibility results were identical for the Δ*vexBDHK* strain and the ΔRND strain supported the conclusion that the VexB, VexD, VexH, and VexK RND efflux pumps were responsible for *V. cholerae* resistance to bile acids in vitro.

### The VexF and VexM Pumps do not Function in Antimicrobial Resistance *in vitro*

The Δ*vexBDHK* mutant had the same antimicrobial susceptibility profile as the ΔRND mutant for all of the tested antimicrobial compounds, including cholate, deoxycholate, Triton X-100, SDS, erythromycin, Polymyxin B, novobiocin, ampicillin, and penicillin (Table 2 and data not shown). This suggested that neither VexF nor VexM functioned in antimicrobial resistance in vitro.

### Multiple RND Efflux Pumps Contribute to Virulence Factor Production

**Figure 1:**
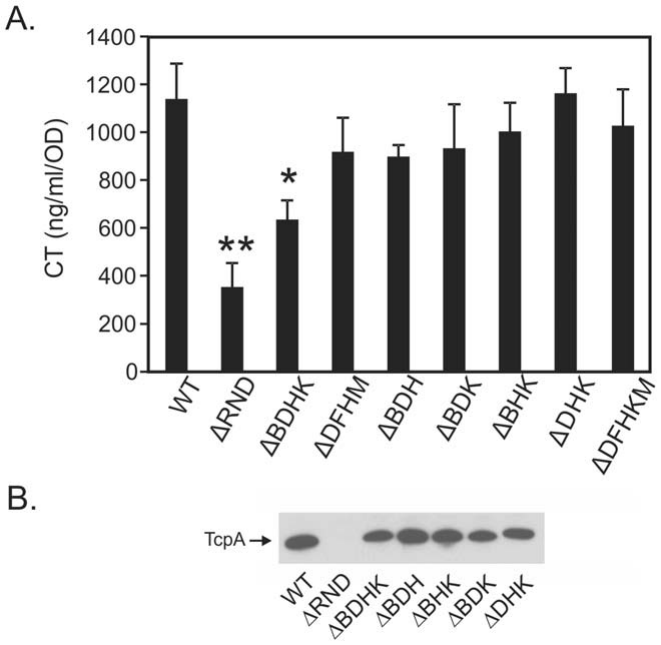
CT and TCP production by RND mutants. CT and TCP production in the indicated strains was determined following growth under AKI conditions. CT (A) and TcpA (B) were detected by CT GM_1_-ELISA and TcpA Western immunoblotting, respectively. Error bars represent the standard deviation of the mean from three or more experiments. Statistical analysis was performed by one-way ANOVA. *p<0.05 compared to wild-type (WT); **p<0.05 compared to all tested strains.

CT production in the *V. cholerae* ΔRND mutant was decreased by ∼70% relative to WT (Fig. 1a) with a corresponding decrease in TCP production (Fig. 1b). This is in agreement with previously reported work [32] and was used as a RND efflux-negative reference for analysis of CT and TCP production by the RND mutant strains generated in this study. CT and TcpA production in the Δ*vexBH*, Δ*vexHK*, Δ*vexDK,* Δ*vexBDH*, Δ*vexBDK*, Δ*vexBHK,* Δ*vexDHK,* Δ*vexDFHM,* and Δ*vexDFHKM* mutants (Fig. 1a, 1b, and data not shown) was not statistically different from WT. In contrast, CT and TcpA production in the Δ*vexBDHK* mutant was reduced by ∼45% relative to WT (Fig. 1a and 1b), suggesting that these four efflux pumps were required for virulence factor production. The observation that the presence of a functional copy of any one of these four RND efflux pumps resulted in a WT phenotype suggested that there is redundancy among these pumps for their function in CT and TCP production. The finding that the Δ*vexBDHK* mutant produced more CT and TcpA than the ΔRND mutant suggested that VexF and/or VexM also contributed to virulence factor production and support the conclusion that at least five of six RND efflux pumps are required for high-level production of CT and TcpA.

### VexH Contributes to *in vivo* Colonization

**Figure 2:**
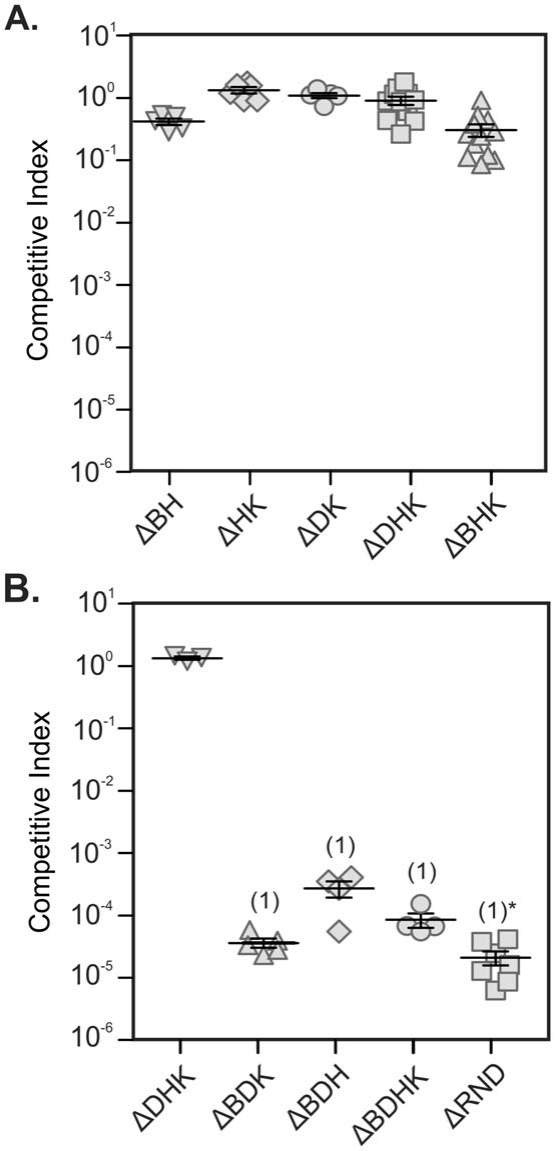
Infant mouse colonization assays with the RND efflux mutants. Competition assays were performed using the infant mouse colonization assay as described in the Materials and Methods. Infant mice were challenged with a ∼10^5^ cfu inoculum containing a mixture of wild-type and the indicated mutant at a ratio of 1∶1 (A) or 1∶100 (B). The competitive index was calculated as the ratio of mutant to wild-type recovered from the small intestine, corrected for the ratio of mutant to wild-type that was present in the inoculum. Each symbol represents one mouse. *The ΔRND mutant was not recovered from mice necessitating the calculation of a theoretical CI as described in the Materials and Methods. Mean and standard deviation are indicated by horizontal bars. Significance was determined using the Mann-Whitney U t-test. (1) p<0.01.

The competitive index (CI) is a measure of fitness of a test strain relative to the WT strain for colonization of the infant mouse small intestine. Mutants that are able to compete equally with the WT strain exhibit a CI of ∼1, whereas mutants that are outcompeted by the WT (i.e. attenuated mutants) will have a CI of <1. Analysis of the mutants constructed in this study showed that the Δ*vexBH,* Δ*vexHK,* Δ*vexDK,* Δ*vexDHK* and Δ*vexBHK* strains competed equally with the WT strain (Fig. 2a). Similar results were previously reported for the Δ*vexBD* strain [32]. In contrast, the Δ*vexBDH* and Δ*vexBDHK* mutants were found to be severely attenuated and could not be recovered from the infant mice when inoculated at a 1∶1 ratio (data not shown). The in vivo attenuation of these mutants did not emanate from an apparent growth defect as all of the mutants competed equally with WT during in vitro competitive growth assays and there were no differences in the growth kinetics of the strains in minimal media (data not shown).

There was a possibility that the detection limit of the infant mouse colonization assay hindered our ability to recover severely attenuated mutant strains (e.g. Δ*vexBDH*, Δ*vexBDHK,* and ΔRND) in the intestinal homogenates. To compensate for this the challenge inoculum was biased for the mutant strains by 100-fold (i.e. 1∶100 ratio of WT to mutant cells) which resulted in an ∼2 log increase in the detection the limit. To validate that the biased input did not affect the CI, we tested the Δ*vexDHK* strain which competed equally with the WT strain at the 1∶1 ratio. The results showed that the Δ*vexDHK* competed equally with the WT strain at the 1∶100 input ratio, confirming that the biased input did not affect the CI value (Fig. 2b). The modified assay was then used to test the Δ*vexBDH*, Δ*vexBDK*, Δ*vexBDHK*, and ΔRND strains. The results of this analysis confirmed the severely attenuated phenotype of each strain (Fig. 2b). However, the Δ*vexBDH* and Δ*vexBDHK* strains, which could not be recovered from infant mice when inoculated at the 1∶1 ratio, were recovered in 30% of the challenged mice using the modified assay. Using data from the colonized mice, the Δ*vexBDH* and Δ*vexBDHK* strains had CI’s that were reduced by 3.7 and 4.1 log units (Fig. 2B). The ΔRND strain still could not be recovered from the mice which is consistent with this mutant having the greatest colonization defect with a >4.8 log reduction in its CI.

**Figure 3:**
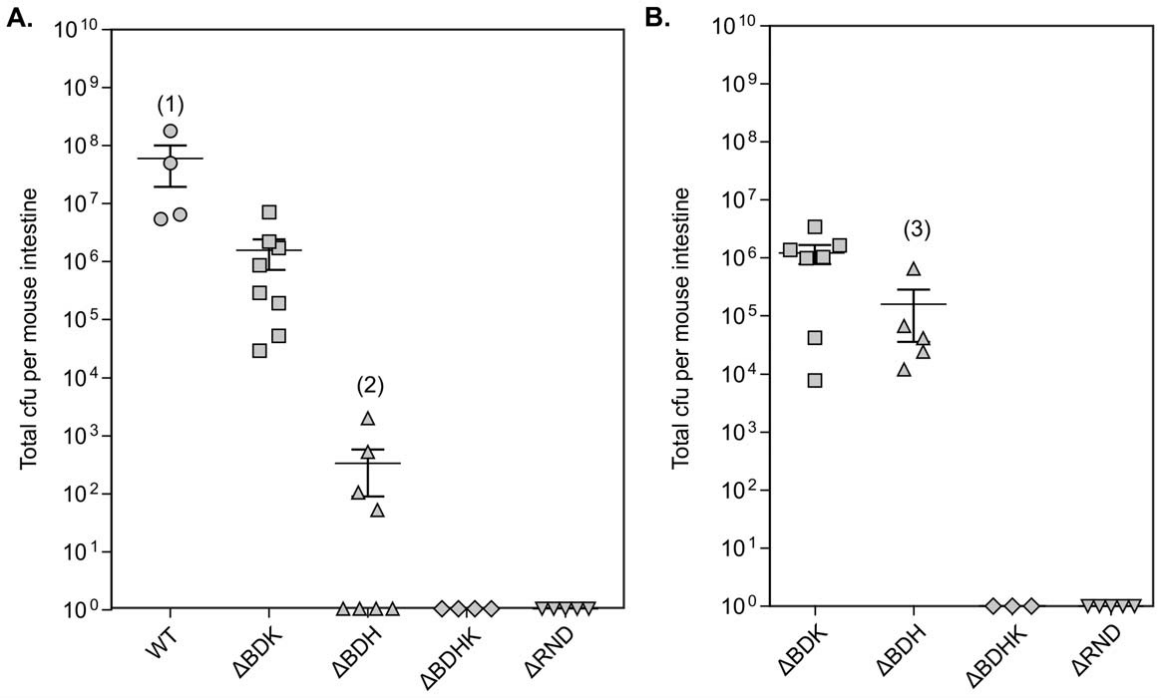
Colonization of the infant mouse small intestine by *V. cholerae* RND efflux mutants. Infant mice were challenged with ∼6×10^6^ cfu (A) or ∼8×10^7^ cfu (B) of the indicated *V. cholerae* mutant. Bacterial loads in the small intestine were assessed after overnight incubation. Means and standard deviation are indicated by horizontal bars. The Mann Whitney U t-test was used to determine significance. (1) p<0.05 compared to all tested strains; (2) p<0.05 compared to Δ*vexBDK*; (3) p<0.05 compared to the ∼6×10^6^ cfu (A) Δ*vexBDH* challenge.

Despite the modifications to the colonization competition assay, the ability to quantify colonization differences between highly attenuated mutants (e.g. the Δ*vexBDH,* Δ*vexBDHK*, and ΔRND strains) was still limited. Therefore, we assessed the ability of these three highly attenuated strains to colonize the infant mouse small intestine in the absence of the WT competitor strain (Fig. 3). Mice were challenged with the mutants at two inoculums: ∼10^6^ cfu/mouse and ∼10^8^ cfu/mouse. The 10^6^ cfu/mouse inoculum was equal to the mutant titer used in the modified competition assay while the 10^8^ cfu/mouse inoculum was used to determine if increasing the challenge dose would facilitate colonization by the mutant strains.

The WT strain and Δ*vexBDK* mutant colonized 100% of the challenged infant mice when administered at 10^6^ cfu/mouse. However, the Δ*vexBDK* mutant exhibited an apparent in vivo growth defect since the mutant replicated to a final in vivo titer was ∼2 log units lower per mouse than was observed with the WT (Fig. 3a). Inoculation of mice with 10^6^ cfu of the Δ*vexBDH* mutant resulted in colonization of about 50% of the challenged mice. In the successfully colonized mice, the Δ*vexBDH* titers were at least 5-logs lower than was observed in mice challenged with the WT strain and 3-logs lower than the output observed in mice challenged with the Δ*vexBDK* mutant (Fig 3a). This indicates that the in vivo growth defect of the Δ*vexBDH* mutant was significantly greater than what was observed for the Δ*vexBDK* mutant. This is also consistent with the severe attenuation of this mutant in the colonization competition assays. When the challenge inoculum was increased to 10^8^ cfu/mouse the Δ*vexBDH* mutant successfully colonized 100% of the challenged mice. In addition, the bacterial titer in each mouse also increased by 3-logs to a level that was equivalent to what was observed with the Δ*vexBDK* mutant (Fig. 3b). The increase in the output titer was limited to the Δ*vexBDH* mutant and was not observed when mice were challenged with 10^8^ cfu of the Δ*vexBDK* mutant. The Δ*vexBDHK* and ΔRND mutants did not successfully colonize the intestinal tract at either inoculum level (Fig. 3a and 3b). This indicates that if either of these strains is able to colonize the infant mouse small intestine, the mutants were present at very low levels that were below our limits of detection. Since we were unable to distinguish an in vivo difference between these two strains, other approaches will be required to assess the function of VexF and VexM in vivo.

## Discussion

Deletion of *vexH* in the WT background did not result in an observable phenotype. There were two plausible explanations for this lack of phenotype: either VexH did not contribute to antimicrobial resistance, or its contribution was masked due to redundancy with one or more of the other five *V. cholerae* RND efflux pumps. The latter was proven true since the introduction of the *vexH* deletion into *V. cholerae* lacking the *vexBDK* RND efflux pumps resulted in increased susceptibility to a number of antimicrobial compounds (Table 2). This showed that VexH possessed a relatively broad substrate specificity that was second only to VexB (Table 2 and data not shown). VexH contributed to cholate, deoxycholate, Triton X-100, novobiocin, and ampicillin resistance, but not to penicillin or erythromycin resistance (which were VexB substrates). Redundant substrate specificity between VexH and VexB is consistent with the observation that VexH has the largest amino acid sequence identity in common with VexB among the *V. cholerae* RND efflux pumps [32]. The *V. cholerae* Δ*vexBDHK* mutant exhibited the same antimicrobial susceptibility profile as the ΔRND strain (Table 2). This suggests that VexB, VexD, VexK, and VexH are the only RND efflux pumps that contribute to antimicrobial resistance in vitro. Although these four RND efflux pumps were redundant for some substrates, they do not have equal activity. All four pumps contributed to bile acid resistance, yet the presence of VexB or VexD is sufficient to provide a WT level of resistance in the absence of VexH and/or VexK (Table 2) [22]. Only in a Δ*vexBD* mutant background can the contribution of VexH and VexK be observed. Together this suggests that VexB and VexD are major contributors to bile acid resistance in vitro, while VexH and VexK have minor roles. This conclusion is likely only relevant to *V. cholerae* grown under the conditions used in our assays as there are reports to suggest that the expression of the RND efflux systems are responsive to environmental cues including those present in vivo in rabbit ileal loops and in humans [22], [38]–[40].

The antimicrobial susceptibility results also suggested that neither VexF nor VexM contributed to antimicrobial resistance in vitro. This was a surprising finding as *vexF* from a non-O1 *Vibro* was reported to produce a functional efflux system when expressed in *E. coli* in conjunction with *V. cholerae tolC* [41]. This discrepancy may reflect strain or functional differences of VexF in a heterologous system [41]. Alternatively, it is also possible that VexF or VexM are expressed under conditions or efflux substrates other than tested in this study (see below).

The finding that *V. cholerae* produces redundant RND efflux pumps that function in bile acid and detergent resistance seems to be an important adaption to facilitate colonization of the small intestine. Redundant bile efflux pumps would provide an obvious benefit since intestinal bile is a natural host defence that microorganisms must overcome in order to colonize the small intestine [1]. Consistent with bile salts being a major substrate of the RND efflux pumps, a number of studies have suggested that bile salts and other components of bile function to induce the expression of the RND efflux systems. In 2004, Chatterjee et. al. [42] reported that *V. cholerae* grown in bile accumulated lower amounts of hydrophobic compounds than *V. cholerae* grown without bile, a phenotype they attributed to bile-dependent induction of active efflux. More recently, we and others have shown that transcription of the *vexAB* and *vexCD* RND efflux systems are upregulated in the presence of bile acids [32], [40]. These results are consistent with the hypothesis that substrates of the individual RND efflux pumps function as effectors to upregulate the expression of the respective RND efflux system. While the chemical effectors that control the expression of *vexH* are unknown, a recent study has suggested that *vexH* expression may be responsive to the iron status of the cell [43]–[45]. This finding, combined with the hypothesized iron limiting conditions *V. cholerae* may encounter late during infection [46], could explain the in vivo induction of VexH in humans during infection [38]. If *vexH* transcription is up-regulated during in vivo colonization as a response to iron availability, then VexH could have a greater role in vivo than indicated by our in vitro analysis.

The function of the RND efflux systems in mediating resistance to host defences is correlated with the ability of many bacterial pathogens to survive, invade, and colonize their hosts [47]–[50]. We therefore expected that *V. cholerae* RND efflux mutants with similar antimicrobial susceptibility profiles would behave similarly in vivo, but our results revealed this to be false. For example, the Δ*vexBDK* and Δ*vexBDH* mutants exhibited similar susceptibility profiles for bile salts and detergents, but the Δ*vexBDH* mutant was more attenuated in vivo than the Δ*vexBDK* mutant and less attenuated than the Δ*vexBDHK* and ΔRND mutants. Consistent with this the Δ*vexBDH* mutant required a two-log higher inoculum than did the Δ*vexBDK* mutant to efficiently colonize the small intestine (10^8^ vs. 10^6^ cfu/mouse, respectively; Fig. 3). This is in contrast to WT which can efficiently colonize when administered at inoculums of 10^3^–10^4^ cfu/mouse [51]. Even at the higher inoculums, neither mutant was able to reach titers in the intestine equivalent to WT. It was noteworthy that administration of the Δ*vexBDH* mutant at 10^8^ cfu/mouse resulted in a three-log increase in the bacterial outputs from the colonized mice relative to inoculation at 10^6^ cfu/mouse. This phenomenon was not observed with the Δ*vexBDK* mutant (Fig. 3b). Together this suggests that the in vivo roles of the RND efflux systems do not completely correlate with their contributions to in vitro antimicrobial susceptibility WT *V. cholerae*. The fact that the Δ*vexBDH* mutant can grow to similar titers as the Δ*vexBDK* mutant when given at a high inoculum suggests that the Δ*vexBDH* mutant may be defective in colonization of the intestinal epithelium.

The intestinal epithelium is covered by a thick mucus layer which provides a diffusion barrier against antimicrobial compounds that are present in the lumen (e.g. bile) [52]. One implication of this is that the epithelial surface likely represents a more amenable environment for growth of antimicrobial hyper-susceptible organisms like the Δ*vexBDK* and Δ*vexBDH* efflux mutants. Thus one possible explanation for the colonization difference observed between the Δ*vexBDK* and Δ*vexBDH* mutants is that they exhibit differential susceptibility to antimicrobial compounds that are present in the intestinal lumen. This idea is supported by the observation that VexH has a broader substrate range than VexK (Table 2) which would make VexH a more important during colonization than VexK. The finding that both VexH and VexK were induced during colonization of the human gut [38] corroborates the idea that these two RND efflux pumps are induced in vivo. Alternatively, it is possible that the colonization differences are due to unknown in vivo growth defects or differential effects on the in vivo induction of the ToxR regulon (see below).

The CT and TCP bioassays showed that VexB, VexD, VexH, and VexK contributed to virulence factor production. However, the Δ*vexBDH*, Δ*vexBDK*, Δ*vexBHK*, and Δ*vexDHK* mutants were not different from WT for CT and TCP production (Fig.1). This suggests that these four efflux systems were functionally redundant for CT and TCP production. Consistent with this result was the finding that VexB was able to complement for the loss of the five other RND efflux systems [32] which was evidenced by the observation that a *vexDFHKM* mutant (which is *vexB+*) was phenotypically identical to WT (Fig. 2). The function of VexF and/or VexM in CT and TCP production was evident as the mutant that lacked *vexBDHK* was attenuated for CT and TCP production, while the mutant that lacked all six RND efflux systems (i.e. ΔRND) produced even less CT and TCP. This observation provides the evidence that VexF and/or VexM are required for WT CT and TCP production. This also indicates that neither VexF nor VexM are able to fully compensate for the loss of the other four RND efflux systems.

Although much is known about how RND efflux systems contribute to antimicrobial resistance, the mechanism of how they affect virulence factor production is not known. We previously showed that the *V. cholerae* RND efflux systems effect on virulence gene expression mapped to *tcpPH* transcription [32], but the connection between RND efflux systems and *tcpPH* transcription has not yet been determined. We hypothesize that the RND efflux systems function to modulate the intra- or extracellular concentration of a low molecular weight molecule that functions as a negative effector of *tcpPH* transcription. Efflux-dependent modulation of an effector molecule represents a mechanism that could be used to link efflux to gene expression. This process could be used to fine-tune the expression of virulence genes in response to the growth environment. For example the efflux of any given effector molecule, which would affect its cellular distribution, would be dependent upon the presence of competing efflux substrates in the bacterium’s growth environment (e.g. components of bile in the GI tract). Consistent with this hypothesis, a number of potential low molecular weight effector compounds have been described in the literature that affect virulence factor production including: fatty acids, bile acids, quorum sensing molecules, cyclic nucleotides, and cyclic peptides [53]–[58]. Significantly, all of these compounds have been reported to be effluxed in Gram negative bacteria [42], [59], [60] which suggests the possibility that effector efflux could be applicable to other bacterial pathogens where the RND efflux systems have also been reported to influence virulence factor production [47], [61]. In addition to negatively affecting *tcpPH* transcription, efflux could also impact genes downstream of *tcpPH* in the ToxR regulon. For example, given the role of the RND efflux systems in bile resistance, it is possible that the loss of efflux could impact intracellular fatty acids pools and thus affect ToxT activity and virulence factor production [62], [63].

In summary, we have shown that VexH contributes to antimicrobial resistance and exhibits broad substrate specificity. VexH was found to be important for intestinal colonization and virulence factor production; phenotypes consistent with *vexH* being in vivo induced in humans [38]. We have also shown that the *V. cholerae* RND efflux pumps have redundant functions, not only in antimicrobial resistance, but also in virulence factor production. Collectively these results support the conclusion that the RND efflux system contribute to *V. cholerae* pathogenesis in two ways. First, the RND efflux systems function to provide the bacterium with protection against antimicrobial compounds that are present in the host. Second, the RND efflux systems are required for efficient production of virulence factors.
